# Regulation of Neuronal Cell Death by c-Abl-Hippo/MST2 Signaling Pathway

**DOI:** 10.1371/journal.pone.0036562

**Published:** 2012-05-09

**Authors:** Weizhe Liu, Junbing Wu, Lei Xiao, Yujie Bai, Aiqin Qu, Zheng Zheng, Zengqiang Yuan

**Affiliations:** 1 State Key Laboratory of Brain and Cognitive Sciences, Institute of Biophysics, Chinese Academy of Sciences, Beijing, China; 2 Institute of Cancer Stem Cell, Dalian Medical University Cancer Center, Liaoning, China; 3 College of Life Sciences, Shandong University, Shandong, China; 4 Beijing Institute of Geriatrics, Xuanwu Hospital of Capital Medical University, Beijing, China; 5 College of Life Sciences, Graduate University of the Chinese Academy of Sciences, Beijing, China; Wayne State University, United States of America

## Abstract

**Background:**

Mammalian Ste20-like kinases (MSTs) are the mammalian homologue of Drosophila hippo and play critical roles in regulation of cell death, organ size control, proliferation and tumorigenesis. MSTs exert pro-apoptotic function through cleavage, autophosphorylation and in turn phosphorylation of downstream targets, such as Histone H2B and FOXO (Forkhead box O). Previously we reported that protein kinase c-Abl mediates oxidative stress-induced neuronal cell death through phosphorylating MST1 at Y433, which is not conserved among mammalian MST2, *Drosophila* Hippo and *C.elegans* cst-1/2.

**Methodology/Principal Findings:**

Using immunoblotting, in vitro kinase and cell death assay, we demonstrate that c-Abl kinase phosphorylates MST2 at an evolutionarily conserved site, Y81, within the kinase domain. We further show that the phosphorylation of MST2 by c-Abl leads to the disruption of the interaction with Raf-1 proteins and the enhancement of homodimerization of MST2 proteins. It thereby enhances the MST2 activation and induces neuronal cell death.

**Conclusions/Significance:**

The identification of the c-Abl tyrosine kinase as a novel upstream activator of MST2 suggests that the conserved c-Abl-MST signaling cascade plays an important role in oxidative stress-induced neuronal cell death.

## Introduction

MST2 shares the highest degree of homology with the *Drosophila* Hippo and plays an important role in apoptotic cell death [1]. Exposure of cells to apoptosis-inducing stimuli such as Staurosporine, Fas ligand, and oxidative stress activates MST family protein kinases. During apoptosis, MST2 was cleaved and underwent irreversible autophosphorylation, which was resistant to phosphatases [2], [3].

It has been shown that MST2 is regulated by Raf-1 through a direct interaction, which prevents dimerization and phosphorylation of the activation loop of MST2 independent of Raf-1's protein kinase activity [4]. RASSF1A (RAS association domain family 1A) causes the disruption of the inhibitory Raf-1 protein from MST2, and releases MST2 to phosphorylate its substrate, LATS1 (the large tumor suppressor 1). MST2 can be co-precipitated with LATS1 only in the presence of Salvador, which synergistically promotes MST2-mediated LATS1 phosphorylation and activation [5]. The activated LATS1 promotes the cytoplasmic translocation of the transcription factor YAP1 (yes-associate protein 1). Moreover, Akt inhibits MST2 activation by phosphorylation at T117 and T384, which leads to inhibition of MST2 cleavage, nuclear translocation, autophosphorylation at T180 and kinase activity [1], [6]. However, the upstream kinase of MST2 during the oxidative stress-induced cell death is largely unknown.

The ubiquitously expressed tyrosine kinase c-Abl is activated by DNA damage agents [7], and c-Abl functions as a transducer of a variety of extrinsic and intrinsic cellular signals including those from growth factors, cell adhesion, oxidative stress and DNA damage [8]. Recently, c-Abl has been linked to oxidative stress-induced neuronal cell death through Cdk5/GSK3β activation and Tau hyperphosphorylation or through p73 upregulation [9]–[11]. STI571, a c-Abl kinase inhibitor, decreases Cdk5 activation and Tau phosphorylation, leading to the inhibition of neuronal cell death [10], [11]. Recently we found that c-Abl phosphorylates and activates MST1 through phosphorylation at Y433 of the c-terminus that stabilizes MST1 through blocking CHIP-mediated proteasomal degradation. This promotes their interaction with the FOXO transcription factors, and thereby induces cell death in neurons [12]. However, there is no conserved tyrosine in the c-terminal motif of MST2 and it is interesting to explore the possibility and molecular mechanism that c-Abl could regulate MST2 in the oxidative stress-mediated neuronal cell death.

In this study, we demonstrate that MST2 is regulated by c-Abl tyrosine kinase. C-Abl phosphorylates MST2 at Y81, which leads to enhancement of MST2 autophosphorylation as well as its homodimerization. Consistently, we found that c-Abl mediated phosphorylation inhibits the interaction between Raf-1 and MST2. The MST2-Y81F mutant, which is unable to be phosphorylated by c-Abl, confers a lower kinase activity and pro-apoptotic ability compared to that of WT MST2. In mammalian neurons, Rotenone, a specific inhibitor of mitochondrial NADH dehydrogenase [12] (complex I), induced MST2 phosphorylation by c-Abl and promotes neuronal apoptosis. Inhibition of c-Abl by using c-Abl RNAi attenuates Rotenone-induced MST2 activation as well as cell death in primary cultured neurons. Taken together, our findings identify a novel upstream kinase of MST2 that regulates the cellular response to oxidative stress.

## Results and Discussion

### c-Abl phosphorylates MST2 at Y81 in vitro and in vivo

**Figure 1:**
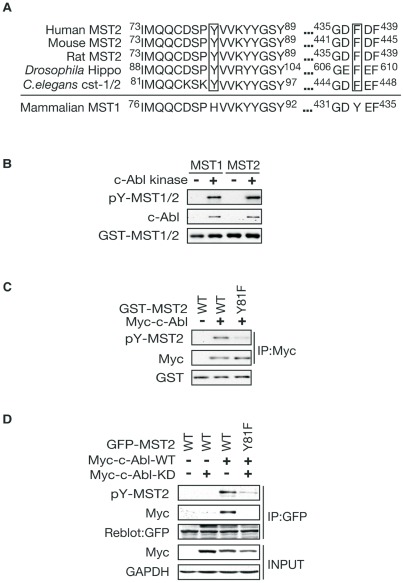
c-Abl Phosphorylates MST2 at Y81 *in vitro* and *in vivo.* (A). Sequence alignment of the mammalian MST2, *Drosophila* Hippo, *C. elegans* cst-1/2 and mammalian MST1. (B). Lysates of HEK 293T cells transfected with Myc-tagged c-Abl or the control vector were immunoprecipitated with anti-Myc antibody and subjected to an *in vitro* kinase assay using full-length GST-MST1 or–MST2 as substrate. Phosphorylation reactions were analyzed by immunoblotting with anti-pan-tyrosine phosphorylation antibody. MST2 and MST1 proteins were tyrosine phosphorylated by c-Abl kinase *in vitro.* (C). *In vitro* kinase assay using the recombinant full-length GST-MST2-WT or–Y81F as a substrate was performed and analyzed as in A. c-Abl phosphorylated MST2 at Y81 *in vitro*. (D). Lysates of HEK 293T cells transfected with FLAG-MST1-WT,–Y81F expression plasmid together with Myc-c-Abl were immunoprecipitated with anti-FLAG antibody and analyzed as in A. Y81 is the phosphorylation site of MST2 by c-Abl *in vivo*.

Previously we found the protein kinase c-Abl mediated oxidative stress-induced MST1 phosphorylation at Y433 [12]. Although it is noted that the phosphorylation site is not conserved in MST1's ortholog, such as MST2 and Hippo (Figure 1A), we found that recombinant GST-fused MST2 as well as MST1 protein was directly phosphorylated by c-Abl by using an *in vitro* kinase assay followed by immunoblotting with an anti-pan-tyrosine antibody (Figure 1B). Sequence analysis revealed that Y81 of human MST2, which is absent in MST1, is conserved among mouse, rat, *Drosophila* (Hippo), and *C. elegans* (cst-1/2, Figure 1A). *In vitro* c-Abl kinase assays using GST-fused MST2 or Hippo as the substrate showed that c-Abl also phosphorylates MST2 and Hippo, indicating there is a conservation of the phosphorylation (Figures S1B and S1C). In addition kinase dead c-Abl failed to phosphorylate MST2 *in vitro* (Figure S1D). Moreover, using mass spectrometry analysis (MS/MS), we found only one phospho-tyrosine residue (Y81) in the immunoprecipitated MST2 from the cells in the presence of c-Abl (Figure S1A). To further confirm that MST2 is a substrate of c-Abl and could be phosphorylated at Y81, we generated the Y81F (Tyrosine to Phenylalanine) MST2 mutation by site-directed mutagenesis. *In vitro* kinase assay showed that the phosphorylation of MST2 Y81F mutant by c-Abl is significantly reduced compared with WT MST2 (Figure 1C). To further validate that c-Abl phosphorylates MST2 at Y81 in cells, the plasmid encoding MST2 WT or Y81F mutant was cotransfected with c-Abl in HEK293T cells. As expected, c-Abl phosphorylated MST2 WT but failed to phosphorylate Y81F mutant in cells (Figure 1D). Taken together, these results support the conclusion that c-Abl kinase phosphorylates MST2 at Y81 within the kinase domain *in vitro* and *in vivo*.

### c-Abl kinase enhances MST2 activation

**Figure 2:**
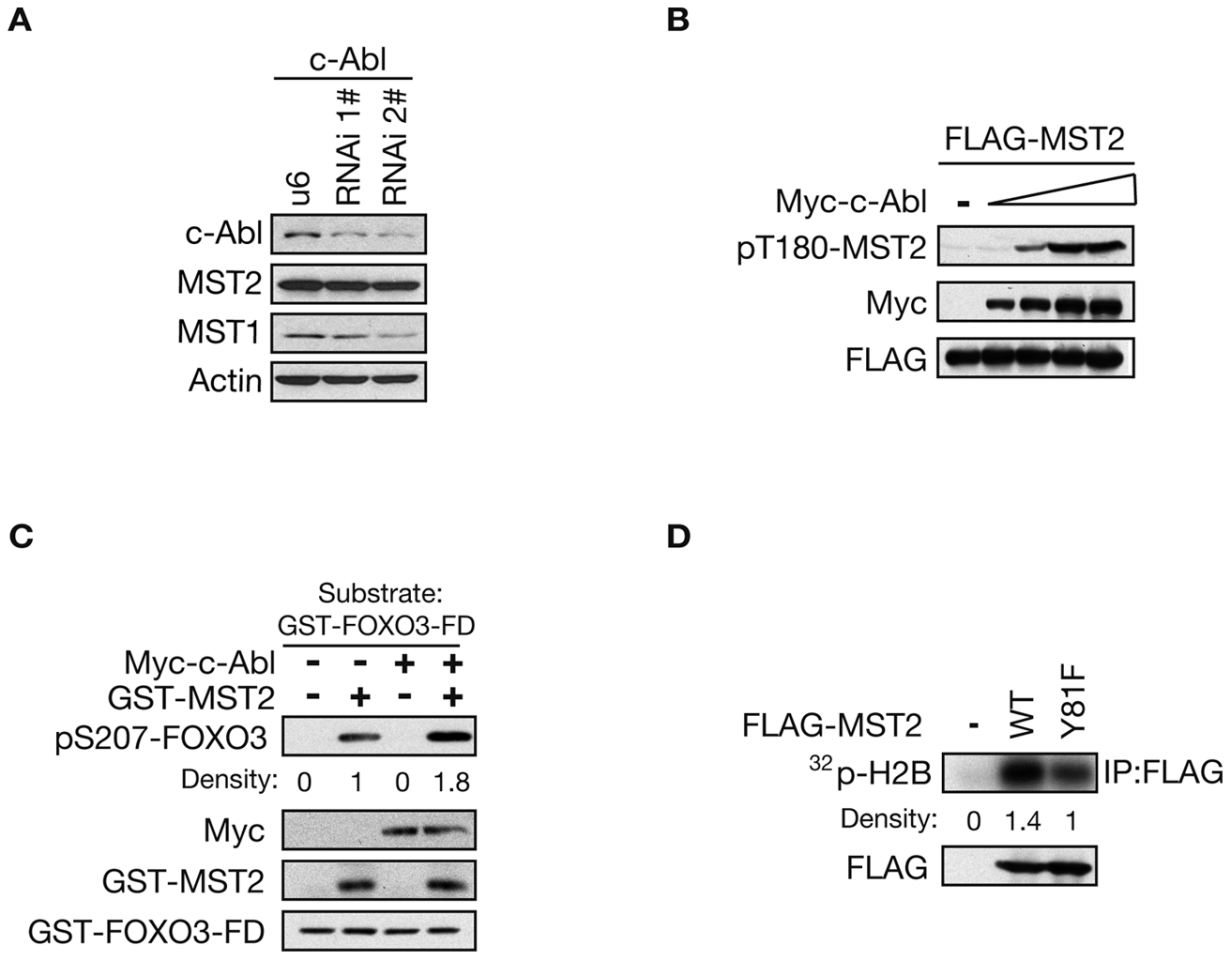
c-Abl Enhances MST2 Kinase Activity. (A). Lysates of Neuro2A cells stably transfected with c-Abl RNAi #1 or #2 or the control vector were immunoblotted with the indicated antibodies. (B). Lysates of HEK 293T cells transfected with FLAG-tagged MST2 alone or together with increasing amounts of Myc-tagged c-Abl expression plasmid were analyzed by immunoblotting with the indicated antibodies. (C). Anti-Myc immunoprecipitates from cells transfected with Myc-c-Abl or the control vector were subjected to the *in vitro* kinase reaction using the recombinant GST-MST2 or GST alone as substrate. GST-MST2 or GST from phosphorylation reactions was then subjected to the second *in vitro* kinase assay using GST-FOXO3-FD as substrate. Phosphorylation reactions were analyzed by immunoblotting with anti-pS207-FOXO3 antibody. The experiments were repeated for three times and quantative density is indicated. (D). Lysates of HEK 293T cells transfected with the FLAG-MST2 or–Y81F expression plasmid were immunoprecipitated with the anti-FLAG antibody and subjected to an *in vitro* kinase assay using Histone H2B as substrate in the presence of [^32^P] ATP. Phosphorylation reactions were analyzed by electrophoresis and autoradiography. The experiments were repeated for three times and quantative density is indicated.

Since we found that c-Abl kinase increases the protein stability of MST1 [12], we next asked whether c-Abl might affect the protein stability of MST2. The expression levels of MST2 are not changed in the absence of c-Abl in comparison with MST1 (Figures 2A and S2). The ability of c-Abl to phosphorylate MST2 within the kinase domain led us next to determine the functional consequences of the tyrosine phosphorylation. HEK 293T cells were transfected with a constant amount of MST2 together with an increasing amount of c-Abl. Immunoblotting analysis revealed that the autophosphoryaltion of MST2, but not the protein levels, increased in direct correlation with the expression levels of c-Abl (Figure 2B). To further delineate the functional interaction between c-Abl and MST2, an *in vitro* MST2 kinase assay was performed and we observed that c-Abl significantly enhanced the kinase activity of MST2 (Figure 2C) by using the recombinant protein of FOXO3 forkhead domain (FD) as the substrate. Correspondingly, we found that c-Abl is capable of enhancing kinase activity of MST2 WT but not Y81 mutant (Figure 2D) by using the Histone H_2_B as the substrate. Thus, the c-Abl-mediated Y81 phosphorylation is essential for MST2 activation.

### c-Abl-mediated phosphorylation of MST2 kinase promotes its homodimerization and disrupts the interaction with Raf-1 proteins

**Figure 3:**
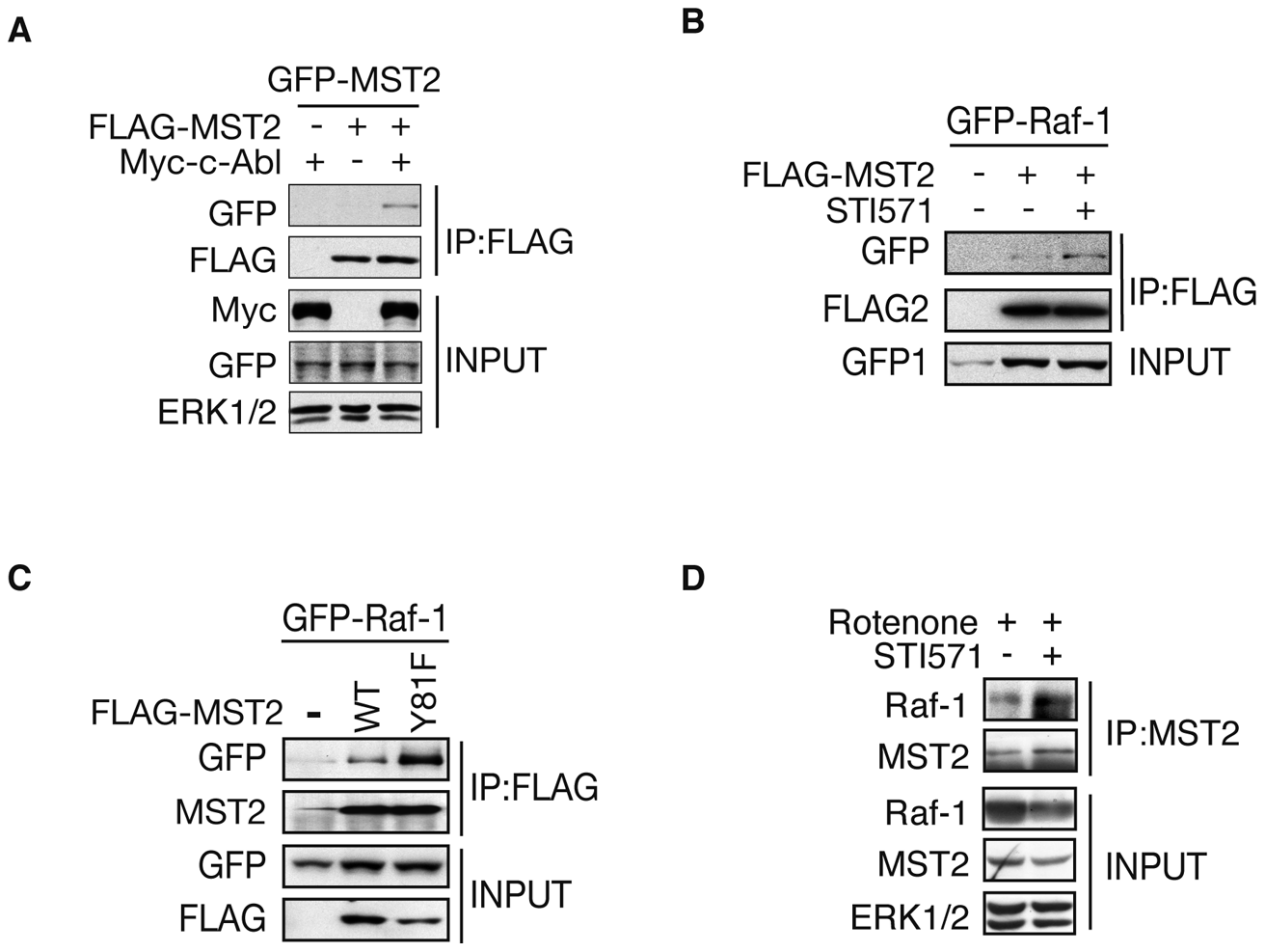
c-Abl-Mediated MST2 Phosphorylation at Y81 Promotes its Homodimerization and Disrupts the Interaction with Raf-1 Proteins. (A). Lysates of HEK 293T cells transfected with GFP-MST2 alone or together with FLAG-MST2 or Myc-c-Abl expression plasmid were immunoprecipitated with FLAG antibody and analyzed by immunoblotting against GFP antibody. (B). HEK 293T cells transfected with GFP-Raf-1 alone or together with FLAG-MST2 expression plasmid were treated with or without 10 µM STI571 for 1 hour. Lysates of cells were immunoprecipitated and analyzed as in (A). (C). Lysates of HEK293T cells transfected with GFP-Raf-1 alone or together with FLAG-MST2-WT or–Y81F expression plasmid were immunoprecipitated with anti-FLAG antibody followed by immunoblotting with the indicated antibodies. (D). MST2 immunoprecipitates from Neuro2A cells treated with or without STI571 were immunoblotted with anti-Raf-1antibody or other indicated antibodies.

Unlike MST1 [12], MST2 is not stabilized by c-Abl-mediated phosphorylation (Figure 2A and S2). We next determined whether c-Abl regulates MST2 kinase activation through a phosphorylation-dependent mechanism. Previous study has shown that phosphorylation of MST1 within the kinase domain by JNK kinase enhances MST1 dimerization and kinase activity [13]. We next examined whether Y81 phosphorylation of MST2 might affect its homodimerization. The co-immunoprecipitation data showed that MST2 homodimerization is enhanced in the presence of c-Abl (Figure 3A) and the Y81F mutant MST2 interacts much less with WT MST2 in the presence of c-Abl (Figure S3), indicating c-Abl-mediated tyrosine phosphorylation enhances the dimerization of MST2 proteins. Raf-1 has been shown to bind to and suppress MST2 by preventing MST2 dimerization in a kinase-independent manner [4]. It raises the possibility that c-Abl might regulate MST2 activation and homodimerization through affecting the interaction between Raf-1 and MST2. C-Abl inhibition with STI571 dramatically increased the interaction between MST2 and Raf-1 (Figure 3B), which led us to investigate whether Y81 phosphorylation of MST2 mediates the interaction between Raf-1 and MST2. As expected, we found that Y81F mutant MST2, but not WT MST2, preferentially binds to Raf-1 (Figure 3C). Furthermore, the endogenous interaction between Raf-1 and MST2 is increased upon STI571 treatment in Neuro2A cells (Figure 3D). Taken together, these results suggest that c-Abl-mediated phosphorylation of MST2 promotes its homodimerization and disrupts the interaction with Raf-1 proteins in an Y81 phosphorylation-dependent manner.

### C-Abl-MST2 signaling mediates Rotenone-induced neuronal cell death

**Figure 4:**
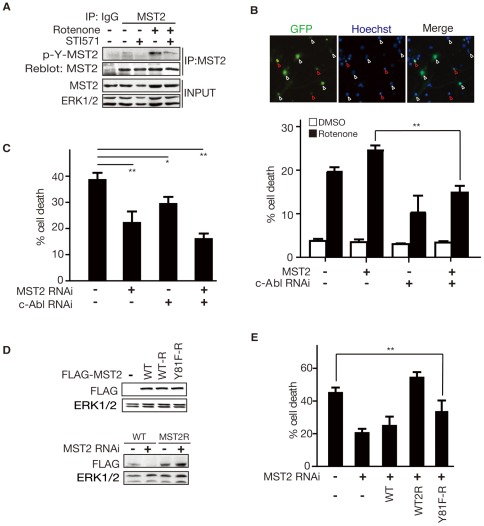
c-Abl-MST2 Signaling Mediates Rotenone-induced Neuronal Cell Death. (A). Neuro2A cells were left untreated or treated with 400 μM Rotenone for 1.5 hours with or without 10 μM STI571. Lysates of cells were immunoprecipitated with anti-MST2 antibody and analyzed by immunoblotting with anti-pan-tyrosine phosphorylation antibody. (B). Cerebellar granule neurons were transfected with the pEGFP alone or together with the FLAG-MST2 expression plasmid, and c-Abl RNAi or control vector as indicated. 72 hours after transfection, neurons were left untreated or treated with Rotenone for 20 hours. Transfected neurons were subjected to immunofluorescence analysis using GFP antibody together with the DNA dye Hoechst 33258 to reveal neuronal nuclei. Representative images of neurons are shown in the upper panel. White arrowheads indicating healthy transfected neurons and red arrowheads indicating transfected neurons that are undergoing apoptosis. The percentage of cell death of GFP-positive neurons is represented as the mean ± SEM. Exposure of MST2-transfected neurons to Rotenone induced neuronal cell death (ANOVA; p<0.01, n = 3), and the cell death was dramatically reduced by c-Abl knockdown (ANOVA; p<0.01, n = 3).(C). Cerebellar granule neurons were transfected with the pEGFP alone or together with the MST2 shRNA, c-Abl shRNA or control vector as indicated. 72 hours after transfection, neurons were treated and analyzed as in B. The percentage of cell death of GFP-positive neurons is represented as the mean ± SEM. Knockdown MST2 or c-Abl protects neurons from Rotenone induced cell death (ANOVA; p<0.01, n = 3). (D). The upper panel shows the expression of the MST2 rescue constructs in Neuro2A cells. Lower panel: Lysates of HEK 293T cells transfected with FLAG-MST2 or FLAG-MST2R expression plasmids together with MST2 RNAi or the control vector, were immunoblotted with the FLAG or ERK1/2 antibody. MST2 RNAi effectively knockdown endogenous MST2 in Neuro2A cells, but not the rescue form of MST2. (E). Cerebellar granule neurons transfected with pEGFP and MST2 RNAi or control vector, alone or together with MST2, MST2R, and MST2R-Y481F expression plasmids, were treated and analyzed as in B. MST2R but not Y81F mutants of MST2R enhanced Rotenone-induced cell death (ANOVA; p<0.01, n = 3). The percentage of cell death in GFP-positive neurons is represented as the mean ± SEM.

We have reported that administration of Rotenone, a mitochondrial complex I inhibitor, led to the activation of c-Abl and sequential transactivation of MST1 [12]. To determine whether tyrosine phosphorylation of MST2 is increased in response to Rotenone, we monitored endogenous MST2 phosphorylation with anti-pan-tyrosine antibody. As shown in Figure 4A, Rotenone treatment stimulates tyrosine phosphorylation of MST2 in Neuro2A cells, which is attenuated by STI571. To determine whether phosphorylation of MST2 by c-Abl in neurons regulate MST2's pro-apoptotic function in response to Rotenone, we employed a plasmid based method of RNA interference (RNAi), which efficiently knock down the endogenous c-Abl (Figure S4). We transfected primary neurons with the FLAG-MST2 alone or together with c-Abl RNAi plasmid, and 3 days after transfection, neurons were left untreated or treated with Rotenone for 24 hours. We found that c-Abl knockdown protects neurons from either Rotenone or MST2 overexpression-induced cell death (Figures 4B and S5). Interestingly, knockdown of MST2 and c-Abl together significantly suppressed neuronal apoptosis (Figure 4C), indicating that c-Abl and MST2 shared a signaling cascade to regulate the neuronal cell death in response to Rotenone treatment. We also observed that STI571 significantly decreased MST2-induced cell death upon treatment with Rotenone (Figure S6). We next defined the significance of c-Abl-mediated phosphorylation of MST2 during Rotenone-induced neuronal cell death. Expression of RNAi-resistant form of MST2 (MST2R), but not WT MST2, reversed the protective function of MST2 RNAi from Rotenone-induced cell death (Figures 4D and 4E). In contrast to MST2R, MST2R-Y81F mutants failed to increase the neuronal cell death in the MST2 knockdown background (Figure 4E). These results indicate that phosphorylation at Y81 is important for MST2-mediated neuronal cell death upon oxidative stress.

In this study, we have discovered an evolutionarily conserved signaling link between the tyrosine kinase c-Abl and the MST family of kinases that mediates responses to oxidative stress in mammalian cells. Our findings generalize the substrates of c-Abl from MST1 to other family members of the MST proteins. Our major findings are: (1) c-Abl phosphorylates MST2 at the conserved Y81 *in vitro* and *in vivo*, (2) the c-Abl-induced phosphorylation of MST2 reduces the interaction between Raf-1 and MST2 and enhances MST2's homodimerization, (3) c-Abl-MST2 signaling plays a critical role in neuronal cell death upon Rotenone treatment. Collectively, we have identified a novel upstream regulator of MST2 underlying the oxidative stress-induced cell death.

The elucidation of the c-Abl-induced phosphorylation of MST2 and consequent disruption of its interaction with Raf-1 proteins provides a molecular basis for how c-Abl kinases activate MST2 signaling in the contexts of oxidative stress in mammalian cells. Previous study has demonstrated that Raf-1 kinase binds to MST2 and prevents its dimerization and autophoshorylation of T180, which results in the inhibition of both MST2 activation and proapoptotic activity [4]. Our findings provide the evidence that c-Abl regulates MST2-Raf-1 complex through Y81 phosphorylation. However, the structural mechanism underlying the disruption of Raf-1 and MST2 association by c-Abl-mediated phosphorylation is still elusive. Furthermore, we also found that c-Abl-induced MST2 phosphorylation at Y81 inhibits the association with Akt (data not shown) indicating that c-Abl mediated phosphorylation of MST2 regulates the interaction between MST2 and its functional partners.

A key conclusion of our study is that the c-Abl-MST signaling link is conserved. MST1 and MST2 are human homologues of Hippo, however, protein sequence similarity between MST2 and Hippo (63.5%) is higher than that of MST1 and Hippo (50%) [1]. Hippo/MST signaling in *Drosophila* and mammals integrates multiple upstream inputs, enabling dynamic regulation of tissue homeostasis in animal development and physiology, especially the organ size control and cell death [14], [15]. Of interest, evidence for *Drosophila* Abl (d-Abl) function was obtained by analysis of mutant phenotypes in the embryonic somatic muscles and the eye imaginal disc. The expression patterns and mutant phenotypes indicate a role for d-abl in establishing and maintaining cell-cell interactions in the developing embryonic muscle and adult eyes [16]. We also found that the recombinant Hippo is phosphorylated by Abl kinase *in vitro* (Figure S2C). Thus, it will be interesting to investigate the conservation and biological functions of c-Abl-Hippo signaling in *Drosophila*.

Our study shows that MST2 possesses a c-Abl phosphorylation site within its kinase domain, which is highly conserved among mammalian (MST2), *Drosophila* (Hippo), and *C. elegans* (cst-1/2), which is absent in mammalian MST1 (Figure 1A). In contrast, the phosphorylation site (tyrosine 433) of MST1 by c-Abl is also absent in mammalian (MST2), *Drosophila* (hippo), and *C.elegans* (cst-1/2) (Figure 1A). We also found that c-Abl activated both MST1 and MST2 and promoted oxidative stress-induced neuronal cell death. Thus, although c-Abl-mediated phosphorylation of both MST1 and MST2 led to enhanced activation of both kinases and might stimulate the same downstream signaling, obviously the regulatory mechanism is different, probably due to the evolutionary diversification. However, whether c-Abl-mediated regulation of MST1 and MST2 plays some specific roles in other circumstances is to be an interesting question in the future studies.

Together with our previous finding [12], the identification of c-Abl signaling to MST kinases further builds the case that c-Abl is a key regulator in neuronal cell death. It will be important in future studies to determine the role of these pathways in the pathogenesis of neurological diseases.

## Materials and Methods

### Plasmids and transfection

The plasmids used were as follows: pCMV-Myc-c-Abl was a gift from Dr. Cheng Cao (Beijing Institute of Biotechnology). MST2-Y81F and other mutants were generated by site-directed mutagenesis. All mutations were verified by sequencing. Raf-1 were cloned into pEGFP-C2 vector at Eco RI and Kpn I restriction sites from the HeLa cDNA library. Mammalian RNAi constructs were designed as described [12]. The hpRNA targeting sequences used include MST2 hpRNA: GGAATATTCTCCTCAATAC; c-Abl hpRNA#1, GACCAACCTGTTCAGCGCT;c-Abl hpRNA#2, AAGCAGCTCGATGGACCTCCA; MST2 Rescue plasmids were generated by creating three silent base-pair mutations in the WT or mutation sequences. Unless stated otherwise, all transfections were carried out in complete medium with Lipofectamine 2000 (Invitrogen) or Vigofect (Vigorous) according to the manufacturer's protocols.

### Tissue Culture

Neuro2A and HEK 293T cells [12], [13] were cultured at 37°C and 5% CO_2_ in DMEM supplemented with 10% fetal bovine serum. DMEM and fetal bovine serum were purchased from Invitrogen. Cerebellar granule neurons were prepared from postnatal day 6 (P6) rat pups [17], [18]. For RNAi experiments, cultures from P6 in vitro (DIV) were transfected with the RNAi or control U6 plasmid together with pEGFP plasmid. After 3 days, cultures were left untreated or were treated with Rotenone for 24 hr. After fixation, the cells were subjected to cell death analysis as described [17], [18]. Briefly, cell survival and death were assessed in GFP-expressing neurons based on the integrity of neurites and nuclear morphology as determined by the DNA dye bisbenzimide (Hoechst 33258). Cell counts were carried out in a blinded manner and analyzed for statistical significance by ANOVA followed by Fisher's PLSD post hoc test. Approximately 200 cells were counted per experiment. All transfections were done by a calcium-phosphate method as described [17], [18].

### Immunoblotting, immunoprecipitation, in vitro kinase Assays and Immunofluorescence

The antibodies used were MST2, c-Abl, phospho-MST1 (Thr183)/MST2 (Thr180), and ERK1/2 (Cell Signaling Technology); GST (Santa Cruz Biotechnology); FLAG-M2 (Sigma); phosphor-tyrosine p-Tyr (4G10) (Millipore); GFP and phosphor-FOXO3 (Ser 207) (Invitrogen). Immunoprecipitations and immunoblotting were carried out as described [12]. Cells were lysed in a buffer containing 20 mM Tris HCl, pH 7.5, 150 mM NaCl, 10% (v/v) glycerol, 1% Nonidet P-40, 2 mM Phenylmethylsulfonyl Fluoride (PMSF), 2 µg/ml Aprotinin and Leupeptin, 2 mM Benzamidine, 20 mM NaF, 10 mM NaPPi, 1 mM Sodium Vanadate, and 25 mM β-glycerophosphate. Lysates were centrifuged at 12,000 g for 15 min at 4°C prior to immunoprecipitation or Western blotting. Aliquots of the cell lysates were analyzed for protein expression and enzyme activity. For immunoprecipitation, lysates were pre-cleared with protein A-protein G (2∶1) agarose beads at 4°C for 60 min. Following the removal of the beads by centrifugation, lysates were incubated with appropriate antibodies in the presence of 10 µl of protein A-protein G (21) agarose beads for at least 1 hour at 4°C. The immunoprecipitates were subjected to *in vitro* kinase assay or Western blotting analysis. Protein expression was determined by probing Western blots of immunoprecipitates or total cell lysates with the appropriate antibodies as noted in the figure legends.

*In vitro* kinase assays were carried out as described [12]. Briefly, immunoprecipitated c-Abl kinase (Millipore) was incubated in the following reaction conditions: 100 mM Tris (pH 7.4), 20 mM MgCl_2_, ATP, 1 µg of GST-MST2 or GST-MST2 mutation as substrate. Immunoprecipitated MST2 from cells was incubated with 0.4 µg of GST-FOXO3-FD or Histone H_2_B in a reaction buffer containing 30 mM Tris (pH 7.4), 20 mM MgCl_2_, 1 mg/ml BSA, ATP. Kinase reactions were separated by SDS-PAGE gel electrophoresis and analyzed by autoradiography or by immunoblotting with indicated antibody.

Immunofluorescence and cell death assay were carried out as described [12]. Freshly fixed neurons were first washed with PBS three times and blocked with 20% goat serum in PBS containing 0.2% triton X-100 to reduce nonspecific antibody binding. Neurons were then incubated with the GFP antibody at 4°C overnight. After washing with PBS three times, Alexa Fluor 488 conjugated secondary antibody (Invitrogen) was used to detect the signal. The secondary antibody was incubated at room temperature for 1 hour and then nuclear morphology visualized using the DNA dye Hoechst 33258 (Sigma) under Zeiss Imager D1 microscope.

### Statistical analysis

Statistical analysis of the data was performed with one-way ANOVA followed by Fisher's PLSD post hoc test using Origin software (Version 8). Data are presented as the mean ± SEM and the number of experiments is indicated in each figure. *P<0.05 or **P<0.01 denotes statistical significance.

## Supporting Information

Figure S1 **Mass spectrum analysis of MST2 phosphorylation by c-Abl kinase.** (A). Immunoprecipitate complex from Figure 1D were subjected to SDS-PAGE followed by Coomassie Blue staining. The band corresponding to MST2 was excised from the gel and digested with trypsin. Phosphorylation sites were mapped by microcapillary liquid chromatography-MS/MS. A phospho-peptide consistent with phosphorylation at Y81 was identified. (B&C). *In vitro* c-Abl kinase assay was performed using GST-Hippo or –MST2 as substrate. Phosphorylation reactions were analyzed by immunoblotting with anti-pan-tyrosine phosphorylation antibody. Both MST2 and Hippo proteins were tyrosine phosphorylated by the recombinant active c-Abl kinase *in vitro*. (D). Lysates of HEK 293T cells transfected with pCMV vector, Myc-c-Abl WT, or Myc-c-Abl KD (kinase dead form) were immunoprecipitated with anti-Myc tag to purify the kinases and then in vitro c-Abl kinase assay was performed using GST or GST-MST2 as substrate. The result was analyzed by immunoblotting with anti-pan-tyrosine phosphorylation antibody (p-Y). GST was taken as a negative control. MST2 could be tyrosine phosphorylated by c-Abl WT but not kinase dead c-Abl. Asterisks (*) indicated non-specific bands.
(TIF)

Figure S2 **C-Abl knockdown does not alter the MST2 protein levels.** Neuro2A cells were transfected with c-Abl RNAi or the control vector. 72 hours after transfection, cells were treated with 50 μg/ml Cycloheximide (CHX) for different time periods. Equal amounts of total protein lysates were subjected to immunoblotting with the MST2 or c-Abl antibody.
(TIF)

Figure S3 **C-Abl-mediated Y81 phosphorylation is important for the dimerization of MST2.** Lysates of HEK 293T cells transfected with GFP-MST2 alone or together with FLAG-MST2 WT or Y81F or Myc-c-Abl expression plasmid were immuno-precipitated with FLAG antibody and analyzed by immunoblotting against GFP antibody.
(TIF)

Figure S4 **C-Abl RNAi efficiently knocks down the endogenous c-Abl in CGNs.** Immunocytochemical analysis of rat cerebellar granule neurons (CGN) transfected with β-galactosidase expression plasmids together with the c-Abl shRNA or control U6 plasmid (in a ratio of 1∶3). C-Abl RNAi reduced endogenous c-Abl expression in b-galactosidase positive cells.
(TIF)

Figure S5 **Representative pictures of cell death in CGNs under rotenone treatment.** CGNs transfected with c-Abl RNAi plasmid or control vector (pBabe/U6), MST2 expressing plasmid or its control vector, together with GFP vector were treated with Retenone (120 nM) for 24 hours. Yellow arrowhead stands for the healthy neurons and red arrowhead indicates apoptotic cells.
(TIF)

Figure S6 **C-Abl inhibitor STI571 significantly decreases MST2 expression-induced cell death.** CGNs transfected with Flag-MST2 plasmid or control vector (pCMV5) together with GFP vector were treated with Retonone only (120–150 nM) or with Rotenone (120–150 nM) and c-Abl inhibitor STI571 (5 μM) for 24 hours. Under Rotenone treatment, MST2 expression increases neuronal death significantly, while the effect could be reversed by STI571 (ANOVA followed by Fisher's PLSD post hoc, p<0.01, n = 3).
(TIF)
