# Supplementary figures and images for: Regulation of Neuronal Cell Death by c-Abl-Hippo/MST2 Signaling Pathway
Source: PLoS One. 2012 May 9;7(5):e36562. doi: 10.1371/journal.pone.0036562 (PMC3348883; doi:10.1371/journal.pone.0036562)

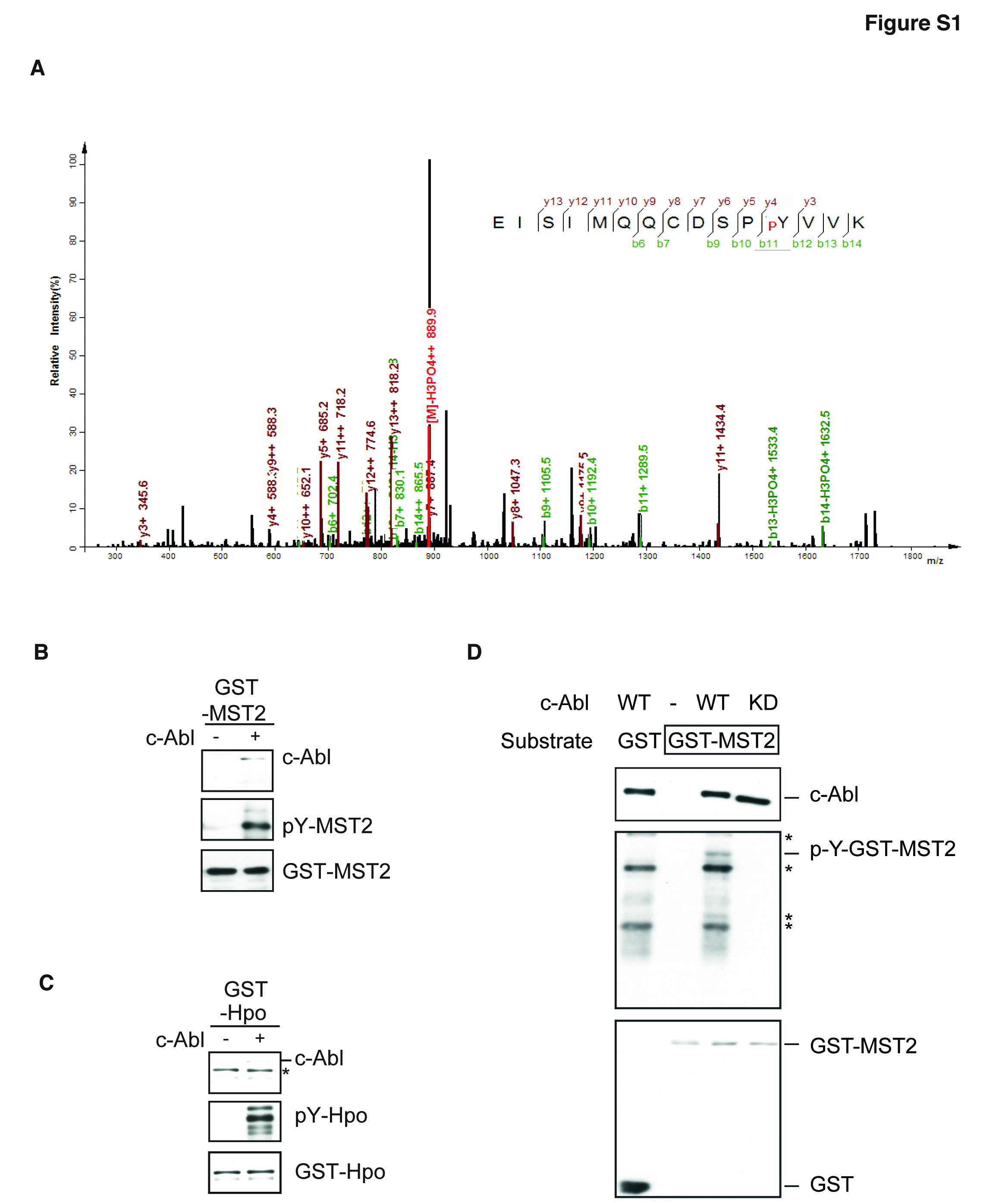

Supplement: Figure S1 — Mass spectrum analysis of MST2 phosphorylation by c-Abl kinase. (A). Immunoprecipitate complex from Figure 1D were subjected to SDS-PAGE followed by Coomassie Blue staining. The band corresponding to MST2 was excised from the gel and digested with trypsin. Phosphorylation sites were mapped by microcapillary liquid chromatography-MS/MS. A phospho-peptide consistent with phosphorylation at Y81 was identified. (B&C). In vitro c-Abl kinase assay was performed using GST-Hippo or –MST2 as substrate. Phosphorylation reactions were analyzed by immunoblotting with anti-pan-tyrosine phosphorylation antibody. Both MST2 and Hippo proteins were tyrosine phosphorylated by the recombinant active c-Abl kinase in vitro. (D). Lysates of HEK 293T cells transfected with pCMV vector, Myc-c-Abl WT, or Myc-c-Abl KD (kinase dead form) were immunoprecipitated with anti-Myc tag to purify the kinases and then in vitro c-Abl kinase assay was performed using GST or GST-MST2 as substrate. The result was analyzed by immunoblotting with anti-pan-tyrosine phosphorylation antibody (p-Y). GST was taken as a negative control. MST2 could be tyrosine phosphorylated by c-Abl WT but not kinase dead c-Abl. Asterisks (*) indicated non-specific bands. (TIF) [file pone.0036562.s001.tif]

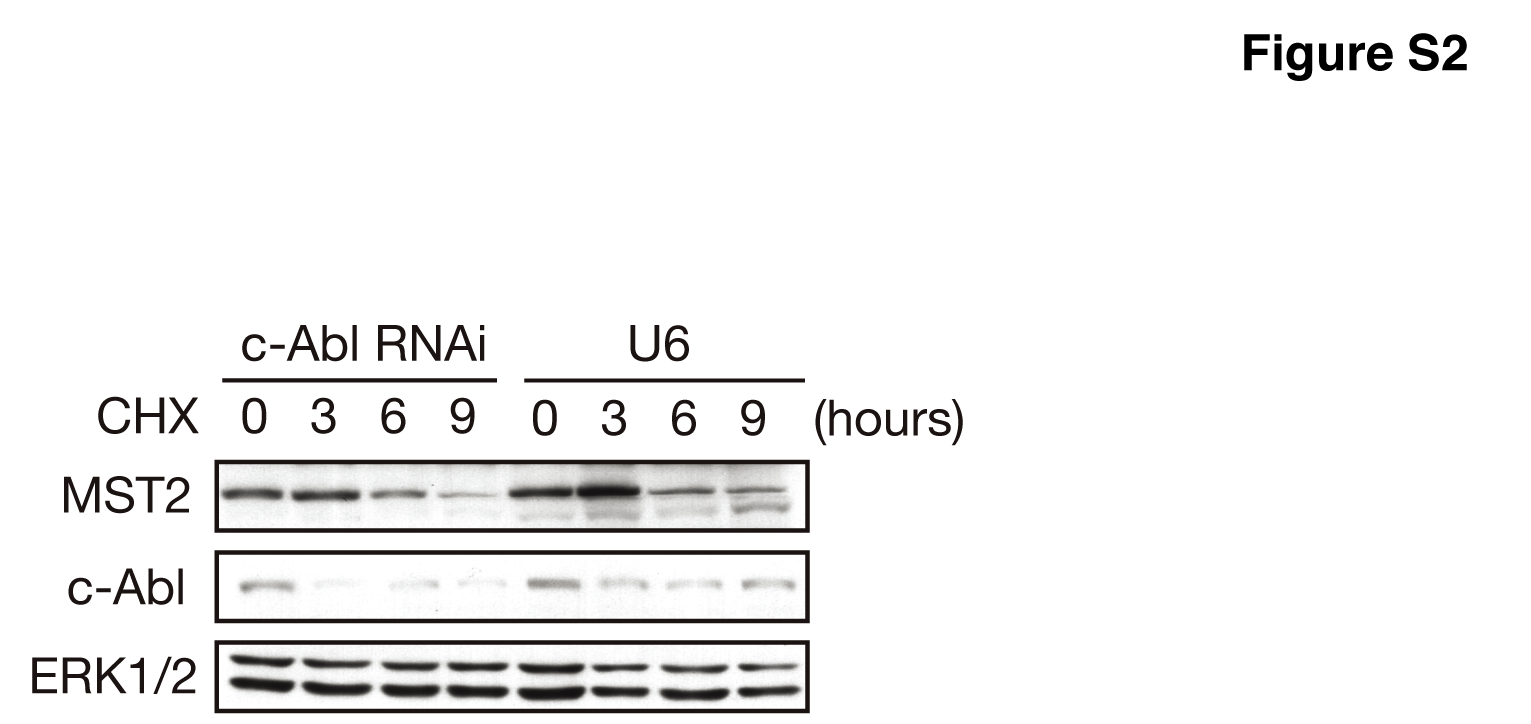

Supplement: Figure S2 — C-Abl knockdown does not alter the MST2 protein levels. Neuro2A cells were transfected with c-Abl RNAi or the control vector. 72 hours after transfection, cells were treated with 50 μg/ml Cycloheximide (CHX) for different time periods. Equal amounts of total protein lysates were subjected to immunoblotting with the MST2 or c-Abl antibody. (TIF) [file pone.0036562.s002.tif]

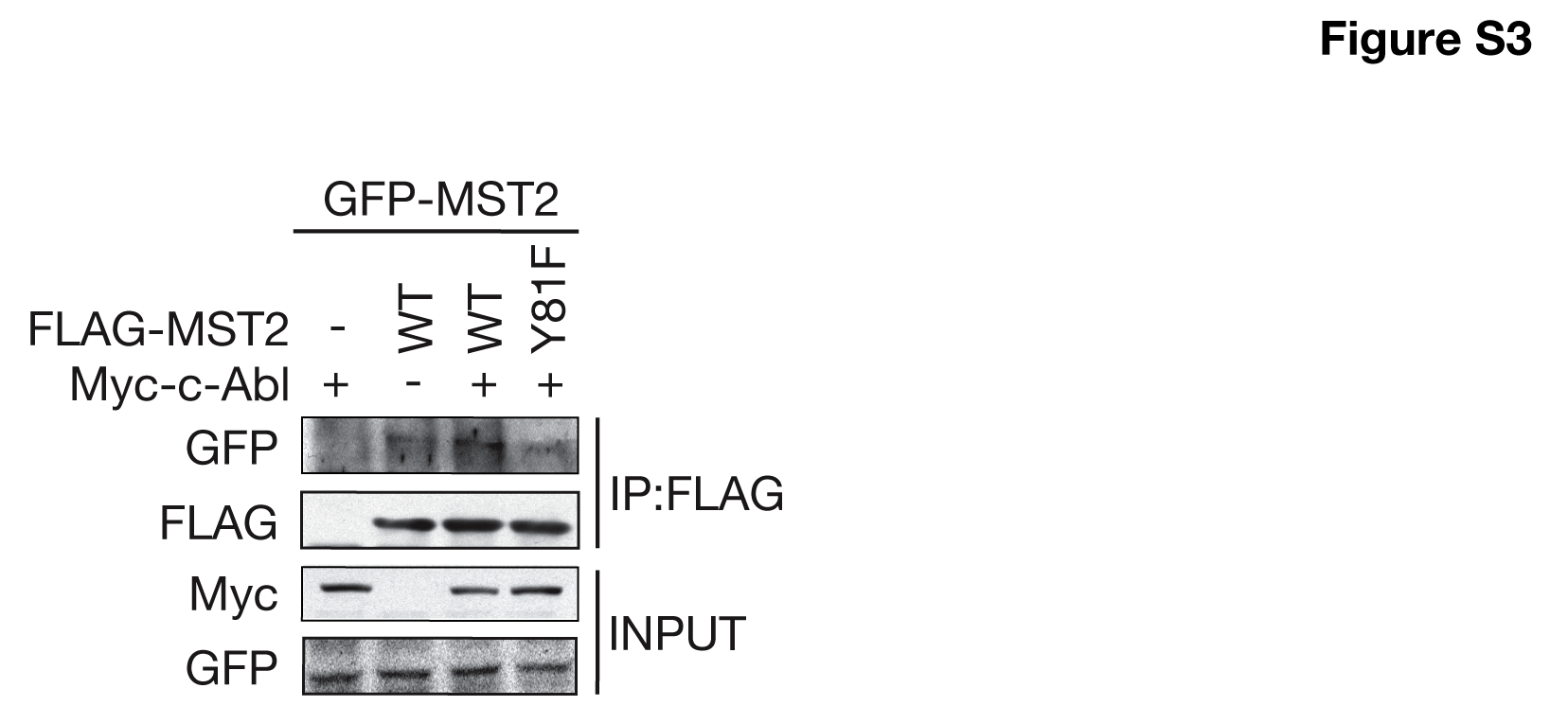

Supplement: Figure S3 — C-Abl-mediated Y81 phosphorylation is important for the dimerization of MST2. Lysates of HEK 293T cells transfected with GFP-MST2 alone or together with FLAG-MST2 WT or Y81F or Myc-c-Abl expression plasmid were immuno-precipitated with FLAG antibody and analyzed by immunoblotting against GFP antibody. (TIF) [file pone.0036562.s003.tif]

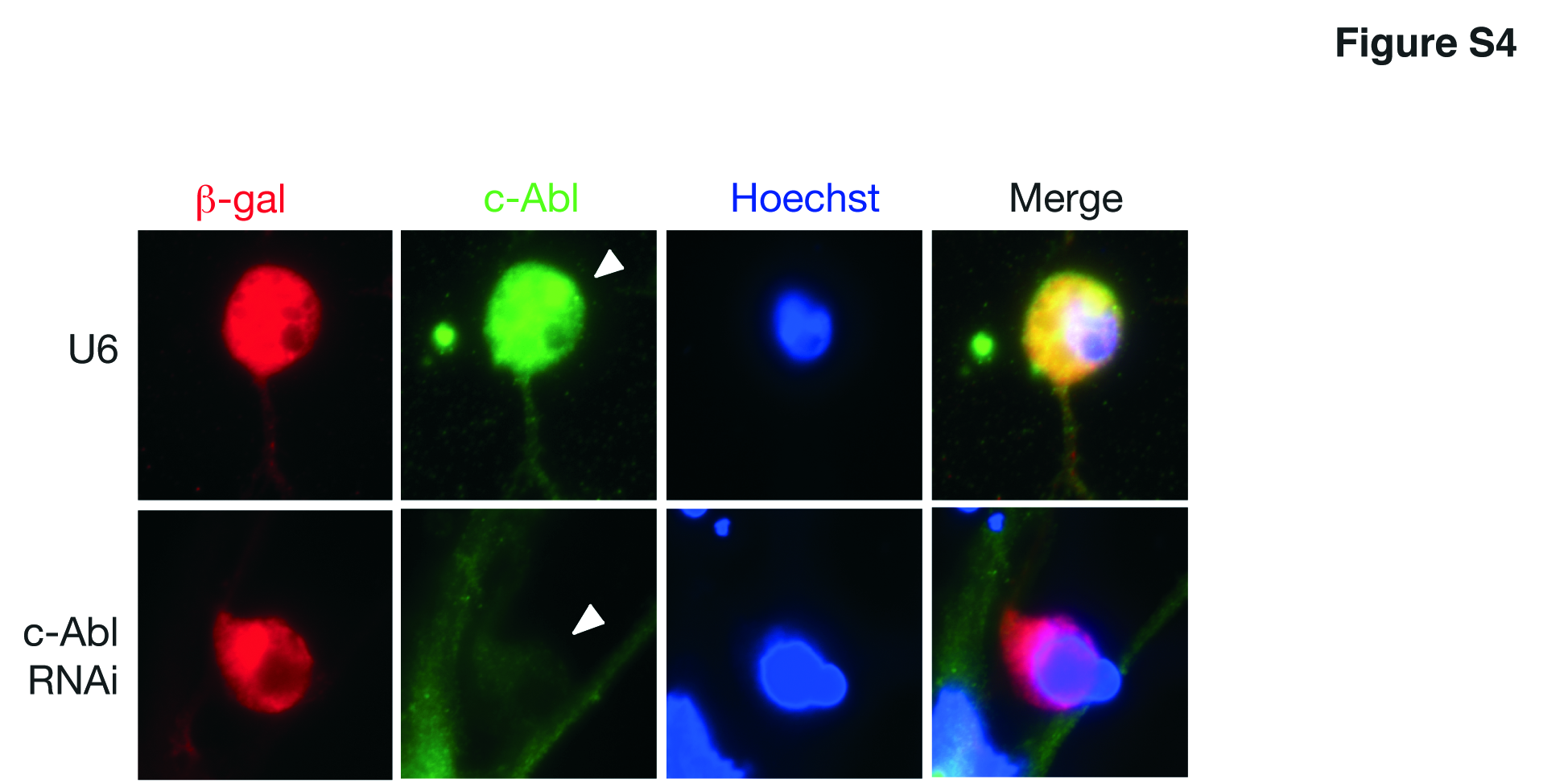

Supplement: Figure S4 — C-Abl RNAi efficiently knocks down the endogenous c-Abl in CGNs. Immunocytochemical analysis of rat cerebellar granule neurons (CGN) transfected with β-galactosidase expression plasmids together with the c-Abl shRNA or control U6 plasmid (in a ratio of 1∶3). C-Abl RNAi reduced endogenous c-Abl expression in b-galactosidase positive cells. (TIF) [file pone.0036562.s004.tif]

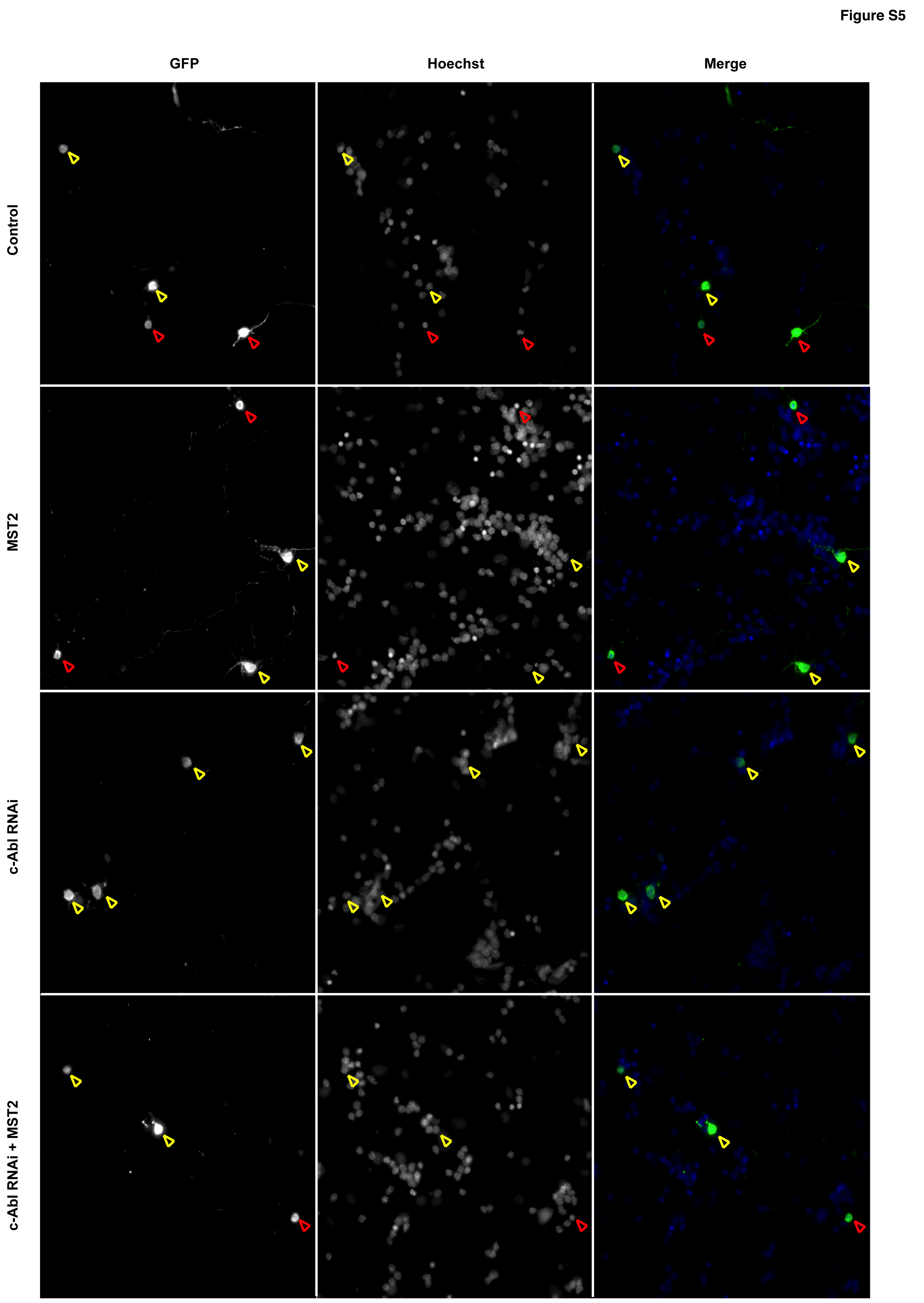

Supplement: Figure S5 — Representative pictures of cell death in CGNs under rotenone treatment. CGNs transfected with c-Abl RNAi plasmid or control vector (pBabe/U6), MST2 expressing plasmid or its control vector, together with GFP vector were treated with Retenone (120 nM) for 24 hours. Yellow arrowhead stands for the healthy neurons and red arrowhead indicates apoptotic cells. (TIF) [file pone.0036562.s005.tif]

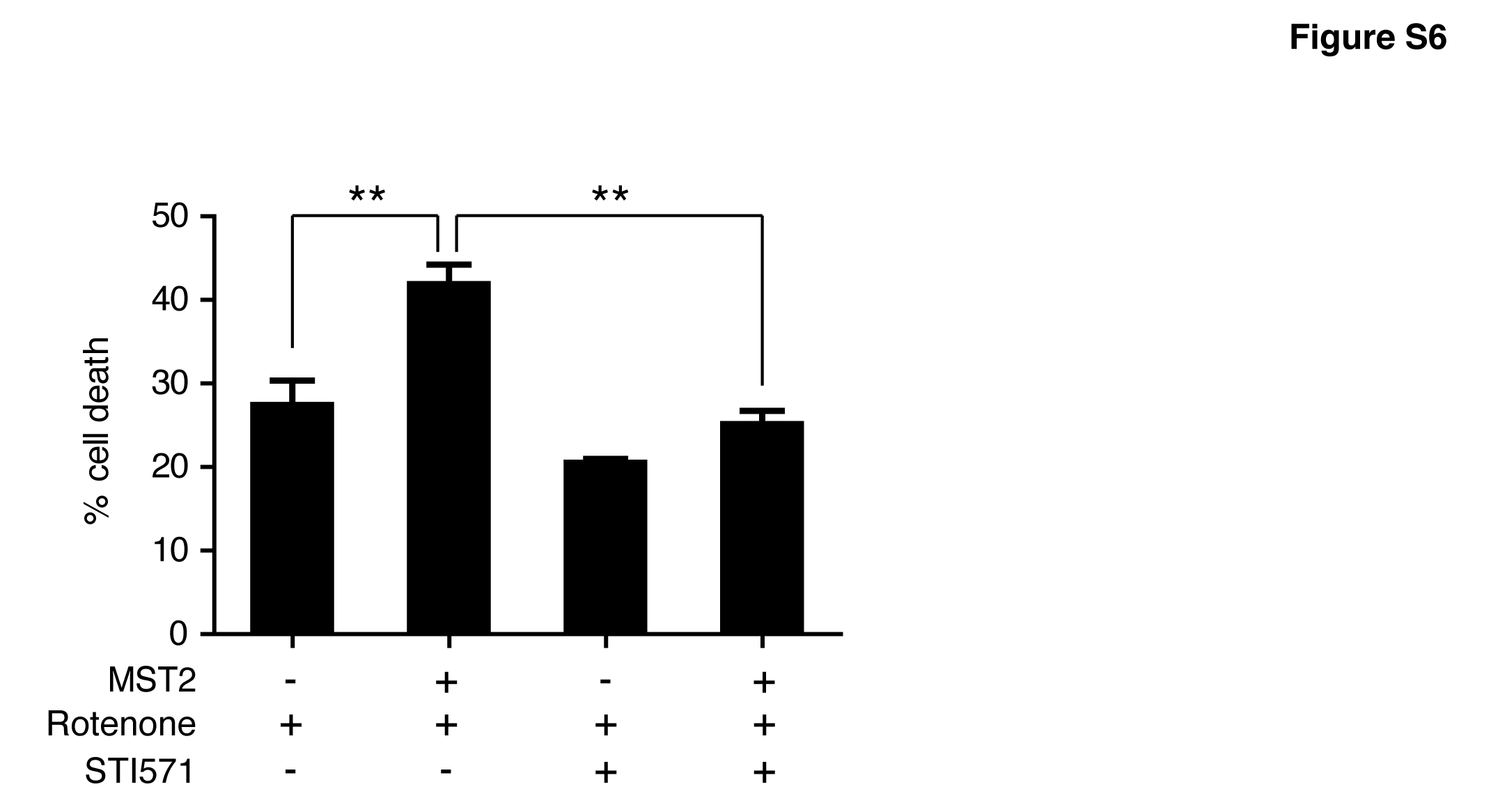

Supplement: Figure S6 — C-Abl inhibitor STI571 significantly decreases MST2 expression-induced cell death. CGNs transfected with Flag-MST2 plasmid or control vector (pCMV5) together with GFP vector were treated with Retonone only (120–150 nM) or with Rotenone (120–150 nM) and c-Abl inhibitor STI571 (5 μM) for 24 hours. Under Rotenone treatment, MST2 expression increases neuronal death significantly, while the effect could be reversed by STI571 (ANOVA followed by Fisher's PLSD post hoc, p<0.01, n = 3). (TIF) [file pone.0036562.s006.tif]
